# Enhanced Systemic and Mucosal Immune Responses to *Haemophilus parasuis* by Intranasal Administration of Lactic-Co-Glycolic Acid Microspheres

**DOI:** 10.3390/vaccines12101103

**Published:** 2024-09-26

**Authors:** Tianyu Lei, Tingting Dai, Liyun Zhuang, Yiting Liu, Xiaohua Li, Cuiqin Huang, Xintian Zheng

**Affiliations:** 1 College of Life Sciences, Longyan University, Longyan 364000, China; 2 Fujian Provincial Key Laboratory of Preventive Veterinary Medicine and Veterinary Biotechnology, Longyan 364000, China

**Keywords:** *Haemophilus parasuis*, PLGA, vaccine, adjuvant, mucosal immune, intranasal

## Abstract

Swine Glasser’s disease, instigated by *Haemophilus parasuis* (*H. parasuis*), is a significant bacterial infection that causes substantial economic losses in pig farming operations. The role of mucosal immunity is pivotal in defending against *H. parasuis*. This study focused on the construction of PLGA microspheres that encapsulate the outer membrane protein OMP16 from *H. parasuis* (PLGA-OMP16) and evaluated their immunological effectiveness in a mouse model. After being intranasally immunized twice, the PLGA-OMP16 microspheres effectively induced IgAs in saliva and nasal and lung fluids. The PLGA-OMP16 microspheres also significantly increased the number of anti *H. parasuis* IgGs in serum. Furthermore, the PLGA-OMP16 microspheres triggered elevated levels of IL-2, IL-4, and IFN-γ. The mice vaccinated with PLGA-OMP16 showed a significant reduction in *H. parasuis* burden in the spleen and lungs following bacterial challenge. These results indicate that intranasal immunization using PLGA microspheres is a promising adjuvant delivery system for vaccines targeting *H. parasuis*.

## 1. Introduction

The infection caused by *Haemophilus parasuis* (*H. parasuis*) in pigs is prevalent worldwide in pig farming and has led to considerable economic losses [1]. *H. parasuis* commonly inhabits the upper respiratory tract of healthy pigs. Under stress or compromised immunity, it can cause pneumonia by infecting the lungs or induce systemic diseases by invading various tissues. Clinically, *H. parasuis* is frequently associated with co-infections or secondary infections with pathogens such as porcine circovirus type 2, porcine reproductive and respiratory syndrome virus, and others, which complicate diagnosis, prevention, and control [2,3].

*H. parasuis* exhibits a complex serotype diversity. While 15 serotypes have been identified thus far, approximately 15% to 41% of the strains remain unclassifiable by conventional methods [4]. The mechanism underlying swine Glasser’s disease, caused by *H. parasuis*, remains not completely understood. *H. parasuis* adheres to the host epithelium through trimer autotransporters (VtaAs) [5] and damages the respiratory tract of the piglets’ cilia and mucous membranes. The virulence of *H. parasuis* is attributed to its ability to evade the innate immune response by degrading IgA [6,7], resisting phagocytosis by alveolar macrophages [8], forming biofilms that shield the bacterium from antibody-mediated killing [9,10], and resisting serum complement [11]. As a result, virulent strains persist in the lungs, while non-virulent strains are eradicated by phagocytes, mainly alveolar macrophages [8,12]. Consequently, mucosal immunity is crucial in providing resistance against *Haemophilus parasuis*.

Vaccination remains an indispensable strategy for preventing *H. parasuis* infection, and numerous *H. parasuis* vaccines have been developed [1]. Among these, inactivated vaccines, including both commercial and domestically produced variants, are the most commonly utilized. Inactivated vaccines can effectively protect against homologous strain attacks and significantly reduce mortality rates. However, they typically do not confer cross-protection against infection by heterologous serotypes [13,14].

Attenuated vaccines offer the advantages of low dosages and the ability to provide comprehensive immunity, including humoral, cellular, and mucosal immunity. Nonetheless, there is a risk associated with attenuated vaccines, namely the potential for revirulence and/or dissemination [15].

Given that the majority of pathogens initially invade the body through mucosal surfaces, which are directly exposed to air, water, food, and the environment, vaccines aimed at these primary contact points between host and pathogen are attractive due to their ability to prevent infections at the primary site of pathogen entry [16]. Antigens administered via mucosal pathways not only induce immune responses at the point of administration and nearby sites, but also elicit immune responses on mucosal surfaces at distant sites, which is a phenomenon known as common mucosal immunity [17]. Vaccines delivered through mucosal surfaces demonstrate swift and widespread distribution of antigens and are capable of eliciting protective mucosal, systemic cellular, and humoral responses [18]. Hence, mucosal vaccines show great potential for offering comprehensive protection against pathogens.

However, numerous vaccine formulations necessitate protection from antigen degradation, especially when delivered via mucosal routes. Employing adjuvants is a strategic method to enhance vaccine stability and adsorption, thereby improving antigen delivery across the physiological and chemical barriers in mucosal tissues [19]. Mucosal adjuvants increase the stability of vaccine formulations by enhancing their bioavailability at mucosal sites. This is crucial, as natural defense mechanisms such as pH levels, mucus layers, and tissue movements may otherwise limit interactions with antigen-presenting cells (APCs) or degrade the antigen. Consequently, this factor greatly influences the intensity and longevity of the immune response [20].

Among the various mucosal immune adjuvants currently available, including liposomes, attenuated toxins, nanoparticles, and others [21], biodegradable microsphere delivery systems offer numerous advantages. These include high biosafety, protection of encapsulated antigens, and sustained release of antigens. Polysaccharides, sodium alginate, lactic acid, and poly lactic acid (PLA) and its copolymer, lactic-co-glycolic acid (PLGA), are materials that are frequently employed in the production of biodegradable microspheres [22]. Among the various polymers utilized in formulating polymeric nanoparticles, PLGA has garnered considerable interest owing to its advantageous characteristics [23]. This study aims to develop PLGA microspheres that can encapsulate antigens of *H. parasuis*, which may confer mucosal immunity to protect pigs against *H. parasuis* infection.

## 2. Materials and Methods

### 2.1. Bacterial Culture

The *Haemophilus parasuis* strain LY02 (serotype 5) [24] was cultured at 37 °C in tryptic soy broth (TSB) (Oxoid, Shanghai, China) or on tryptic soy agar (TSA) (Oxoid, Shanghai, China) supplemented with 10 μg/mL of nicotinamide adenine dinucleotide (Oxoid, Shanghai, China) and 5% bovine serum (HyClone, Beijing, China). The animal experiments were conducted in accordance with the guidelines for experimental animals of the Ministry of Science and Technology (Beijing, China). The study protocol was approved by the Ethics Committee of Longyan University (permit number LY2021009x).

### 2.2. Microsphere Preparation

The OMP16 antigen of *Haemophilus parasuis*, expressed by *E. coli*, was prepared by following previously published methods [25]. The PLGA microparticles were fabricated using the double solvent evaporation technique [26]. To summarize, 0.33 g of PLGA (Sigma, Shanghai, China) was dissolved in 10 mL of dichloromethane, while 1 mg of *H. parasuis* OMP16 protein was dissolved in 1 mL of PBS (pH 7.4, 10 mmol/L) and stirred at 800 rpm for 5 min to form the primary emulsion. Subsequently, 50 mL of a 2% (wt%) polyvinyl alcohol (PVA) aqueous solution was added to the mixture and stirred at 800 rpm for another 5 min to achieve a water-in-oil-in-water (W/O/W) complex emulsion. The emulsion was then poured into 100 mL of a 0.3% PVA aqueous solution and stirred at room temperature for 3 h at 1000 rpm. The dichloromethane was evaporated to dryness. After centrifugation at 2500 rpm for 10 min, the microspheres were collected and washed three times with ultrapure water. The microspheres were then suspended in a specified concentration and freeze-dried for 72 h to obtain powder, which was subsequently stored at 4 °C.

### 2.3. Observation of Microsphere Morphology and Determination of Particle Size

The microspheres’ structure was analyzed using a scanning electron microscope (SEM) (Hitachi, S3400 N, Tokyo, Japan). Following dispersion in sodium acetate buffer, a thick layer of conductive platinum was applied using a sputter ion coater. The SEM images were captured at a 15 mm working distance with a 15 keV beam. The particle size distribution was evaluated using a particle size analyzer (Microtrac, S3500, Pennsylvania, PA, USA), and the resulting images were processed using Microtrac-FLEX software (version 11).

### 2.4. Determination of Encapsulation Efficiency and Loading Capacity of PLGA Microspheres

The efficiency of microsphere encapsulation was determined using a previously described extraction technique [27]. A total of ten milligrams of PLGA microspheres, containing OMP16 and 1 mL of 1N NaOH (pH 11.5), was placed in a tube and then on a rotator set at 30 rpm and 37 °C for 12 h. Following this, the samples underwent centrifugation, and the resulting supernatant was employed to measure the antigen concentration using a BCA protein assay kit (Beyotime Biotech, Shanghai, China). The formula for the encapsulation efficiency was as follows: Encapsulation efficiency (%) = (Amount of loaded OMP16/Amount of total OMP16) × 100%. The loading capacity (LC) was determined using the following equation: LC (μg of OMP16/mg microspheres dry weight) = (Total amount of OMP16–Free OMP16)/mg microspheres dry weight.

### 2.5. Animal Vaccination Programs

All animal experiments followed the recommendations in the China Regulations for the Administration of Affairs Concerning Experimental Animals 1988. One hundred sixty-two six-week-old female Balb/c mice (purchased from Minhou County Wu experimental animal trade Co., LTD, Fuzhou, China) were divided into three groups, including the PLGA-OMP16 group, OMP16 group, and PBS group. A sterile polyethylene tube was inserted 0.2 cm into the nostril of a non-anesthetized animal (in the supine position) and sprayed into the nasal cavity to administer the intranasal vaccination. A new dose was administered only when the previous one was fully absorbed. Two weeks later, the booster vaccination was administered at the same dosage as the initial immunization. The vaccine dosage was adjusted as follows: for the PLGA-OMP16 group, each mouse received 10 μL of PLGA microspheres and 30 μg of OMP16; for the OMP16 group, each mouse received 30 μg of OMP16; and for the PBS group, each mouse received 10 μL of PBS. This experiment was independently repeated twice.

### 2.6. Sample Collection

Two-hundred microliters of blood was collected via the retro-orbital vein on days 0, 14, and 28 post-immunization using a 0.5 × 100 mm blood collection needle under ether anesthesia. Subsequently, the serum was stored at −70 °C. The saliva production was induced following the method outlined by Jaganathan and Vyas [28], where mice received an intraperitoneal injection of 0.2 mL of sterile pilocarpine solution (10 mg/mL). The saliva samples were then collected from the mice using a capillary tube after 20 min.

Fourteen days after the booster immunization, 18 mice from each group were euthanized using isoflurane. The spleens were collected aseptically immediately after euthanasia for cytokine detection. The nasal washings were obtained by inserting a cannula into the trachea of euthanized mice and flushing the nasal cavity three times with 0.5 mL of 1% BSA/PBS (pH 7.4). The bronchoalveolar lavage (BAL) washings were collected by flushing the trachea with 1 mL of ice-cold lavage medium (0.9% NaCl, 0.05% Tween 20, 0.1% NaN3, 1 mM PMSF). After the intestinal contents were removed, the small intestine was flushed with 2 mL of PBS containing 0.1% BSA, 50 mM EDTA, and protease inhibitors (0.1 mg of soybean trypsin inhibitor/10 μg of leupeptin per ml) to obtain intestinal wash samples. Subsequently, all nasal, lung, and intestinal washings were freeze-dried, reconstituted in a fixed volume (0.5 mL) of PBS, and stored at −70 °C until further use. Each group consisted of 3 mice, and the experiments were independently conducted twice.

### 2.7. Evaluation of Serum Humoral Response

The humoral immune response was assessed by measuring serum IgG levels using an indirect enzyme-linked immunosorbent assay (ELISA) by following a previously established protocol [25]. In brief, 96-well microtiter plates (Sangon Biotech, Shanghai, China) were coated with OMP16 (100 ng/well). Following an overnight incubation at 4 °C, the unbound proteins were eliminated by washing each well five times with 200 μL of PBS containing 0.05% Tween 20 (PBST). Subsequently, the plates were blocked at room temperature for 2 h using a blocking solution consisting of 5% skimmed milk in PBS, which was followed by three washes with PBST.

Next, the mouse serum samples were diluted 1:80 in PBS containing 1% bovine serum albumin (BSA) (Sangon Biotech, China), and 100 μL of each dilution was added to the appropriate wells in triplicate. After incubating for 1 h at 22 °C, the plates were washed three times with 200 μL of PBST. Subsequently, each well was incubated with 100 μL of horseradish peroxidase (HRP)-conjugated goat anti-mouse IgG (Bioss, Beijing, China) and diluted 1000 times in PBS with 1% BSA for 1 h at 22 °C. The unbound antibodies were removed by washing the plates five times with 200 μL of PBST.

The bound antibodies were visualized by adding 100 μL of 3,3′,5,5′-tetramethyl benzidine dihydrochloride (TMB) (Beyotime Biotech, Shanghai, China) to each well and incubating the plates for 10 min at 22 °C in the dark. The enzymatic reactions were halted by the addition of 50 μL of sulfuric acid (H2SO4) per well, and the optical density (OD) of each well was subsequently assessed at 450 nm using an ELISA microplate reader from Sangon Biotech, China.

### 2.8. Evaluation of Mucosal Immune Response

To examine the mucosal immune response, the levels of secretory antibodies (sIgA) in saliva, nasal, bronchoalveolar lavage (BAL), and intestinal secretions were evaluated. An IgA ELISA kit from Sangon Biotech, China, was used to quantify IgA concentrations in mucosal samples. The ELISA kit used for measuring IgA levels had a detection range from 6.25 to 400 pg/mL. To minimize potential interference, samples were diluted up to 50-fold using the diluent provided in the kit.

### 2.9. Determination Titers of Cytokines

After the mice received booster immunizations for 14 days, the spleens were harvested. Subsequently, the spleens were weighed and homogenized to prepare 10% *w*/*v* homogenates using a cold solution of 1% 3-[(3-cholamidopropyl)dimethylammonio]-1-propanesulfonate (CHAPS) in PBS. The splenocytes were then adjusted to a concentration of 4 × 10^6^ viable cells per ml in RPMI 1640 medium supplemented with 10% fetal calf serum and an antibiotic/antimycotic solution. These cell suspensions were cultured in 24-well plates and stimulated with OMP16 at a concentration of 10 µg/mL or left in medium alone. The cultures were then incubated for 48 h at 37 °C with 5% CO_2_ to induce cytokine expression in vitro. Post-incubation, the supernatants were collected for cytokine quantification using ELISA sandwich assays with mouse IL-2, IL-4, TNF-α, and IFN-γ ELISA kits from Sangon Biotech, China, as per the manufacturer’s protocol. Each group consisted of 3 mice, and the experiments were independently conducted twice.

### 2.10. Challenge Test in Mice

Fourteen days after the second immunization, mice were intraperitoneally injected with 4 × 10^9^ CFU of *H. parasuis* serotype 5, strain LY02. Daily detailed observations were documented until the 7th day following the challenge to monitor clinical signs and mortality. The necropsies were conducted on the mice that died on the same day, as well as on those that survived beyond the 7-day mark, and any pathological alterations with the corresponding time of occurrence were noted.

### 2.11. Bacteriological Analysis

The bacterial analyses of 18 mice per group were conducted 24 h post-challenge. The protocol for bacterial analysis was previously outlined [29]. The spleen and lung tissues were collected under aseptic conditions from each euthanized animal immediately after necropsy. The tissue homogenates were diluted serially with PBS, and 100 μL of each dilution (103-, 104-, and 105-fold) was plated onto selective media, followed by incubation at 37 °C for 36 h. The CFU values per mg of the homogenized tissue were calculated subsequent to confirming *H. parasuis* presence through colony PCR [30].

### 2.12. Statistical Analysis

The statistical significances and differences were analyzed using OriginPro 2018C 64-bit, Northampton, MA, USA, and one-way ANOVA and Dunnett’s post hoc tests were employed for comparison with the control group.

## 3. Results

### 3.1. Morphology Observation and Encapsulation Efficiency of the Microspheres

**Figure 1:**
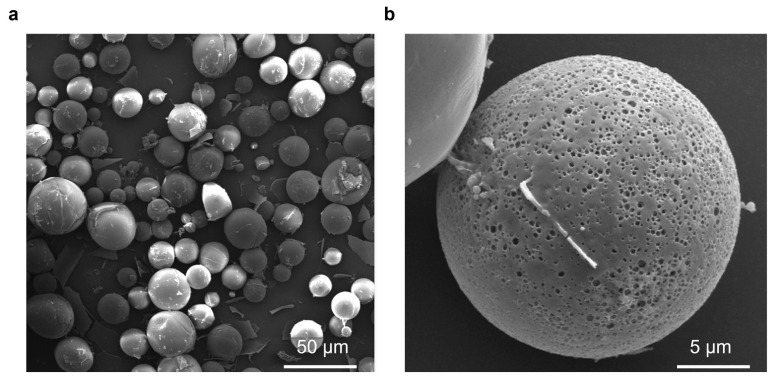
Morphology of the PLGA microsphere. The surface morphology of the PLGA-OMP16 microspheres was visualized using a scanning electron microscope to confirm their spherical structure. The microspheres were found to be spherical and smooth with a size ranging from 10 to 50 μm (**a**). Additionally, small holes distributed on the surface of the microspheres were observed (**b**).

To ensure the microspheres had the desired morphology and uniformity and to confirm their ability to provide stable and predictable antigen release, we conducted SEM observations of the microsphere morphology. The results revealed that the PLGA-OMP16 microspheres exhibited spherical and smooth morphology, which is crucial for the stable delivery and efficacy of the vaccine. The size range was consistently uniform between 10 and 50 μm, meeting the required specifications for use (Figure 1a). The microspheres displayed several small pores on their surface (Figure 1b). The encapsulation efficiency of the microspheres was 81.3% with a loading capacity of 28.93 ± 0.05 g of OMP16 per milligram of the dry microspheres.

### 3.2. Evaluation of Serum Antibody Levels Induced by the Microspheres

**Figure 2:**
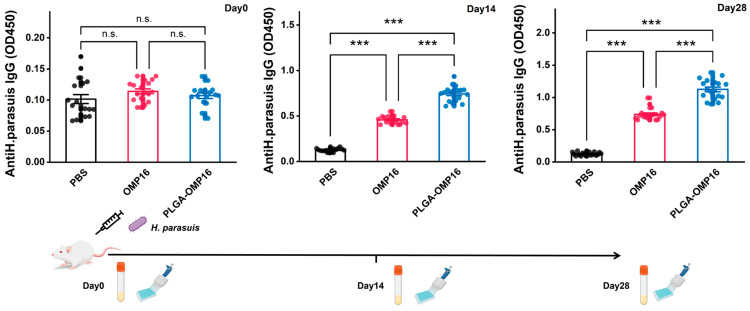
Anti-OMP16 IgG titer in serum after immunization. The serums from the anti-*H. parasuis* IgG profiles of the mice immunized with various formulations were assessed. The values are presented as the mean ± standard deviation (*n* = 18), and the serum samples were collected on days 0, 14, and 28. The statistical significances are denoted by n.s., *p* > 0.05, ***, *p* < 0.001.

The specific antibodies against *H. parasuis* were quantified to assess the humoral immune response of the PLGA-OMP16 microspheres. The findings demonstrated a significant increase in IgG titers following booster vaccination with PLGA-OMP16 and OMP16 compared to primary vaccination. Furthermore, PLGA-OMP16 microspheres induced higher IgG responses than the OMP16 group (*p* < 0.05) (Figure 2).

### 3.3. Evaluation of Mucosal Antibody Concentration

**Figure 3:**
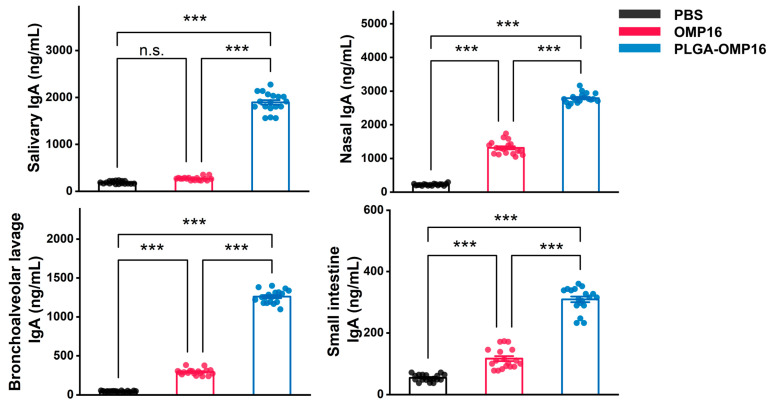
The concentrations of IgAs in mucosal secretions were assessed. Following a 2-week booster dose, the levels of secretory IgAs in nasal, salivary, BAL, and intestinal washes of mice immunized with various formulations were determined. The data are presented as the mean ± standard deviation (*n* = 18). The statistical significances are denoted as follows: n.s., *p* > 0.05; ***, *p* < 0.001.

The mucosal secretions of the mice collected 14 days after the final immunization were evaluated for IgAs to assess mucosal immunity induced by PLGA-OMP16. The results revealed robust detection of IgA antibodies in salivary, nasal, and BAL secretions, with minimal detection in small intestine secretions. Significantly higher IgA concentrations were observed in the PLGA-OMP16 group compared to the OMP16 group (*p* < 0.001) (Figure 3).

### 3.4. Estimation of Cytokine Concentration

**Figure 4:**
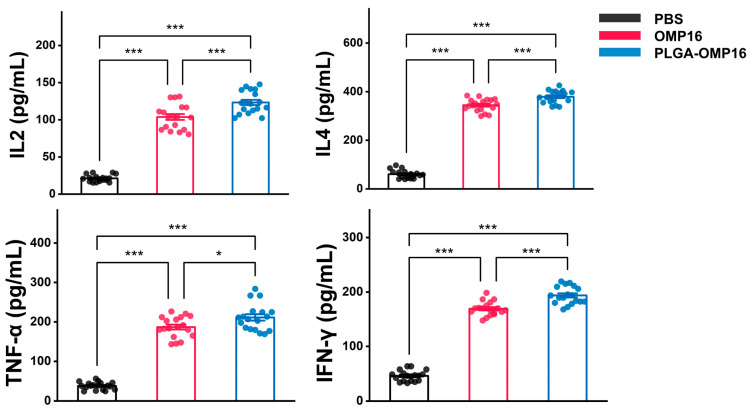
Cytokine analysis was performed using ELISA to quantify the IL-2, IL-4, TNF-α, and IFN-γ levels in the supernatant of splenocytes stimulated in vitro for 48 h. The splenocytes were harvested from mice 14 days after the booster immunization (*n* = 18 mice/group). The statistical significance was denoted as follows: *, *p* < 0.05 ***, *p* < 0.001.

At 14 days post booster immunization, the spleens were collected from three mice in each experimental group for cytokine analysis. The results showed a significant elevation in the concentrations of IL-4, IL-2, IFN-γ, and TNF-α in the PLGA-OMP16 group compared to the other groups (*p* < 0.001) (Figure 4).

### 3.5. Challenge Test

**Figure 5:**
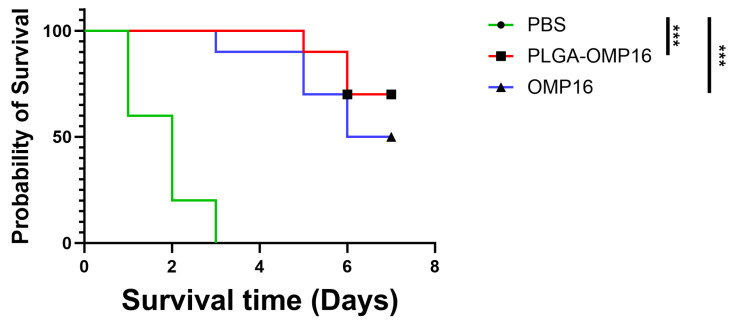
Survival analysis. The mice that received intranasal vaccination twice were subsequently challenged intraperitoneally with 4 × 10^9^ CFU of *H. parasuis* strain LY02 (serovar 5) on day 28 and monitored for 7 days (*n* = 10 mice/group). ***, *p* < 0.001.

To assess the protective immunity, each mouse received an injection of 4×10^9^ CFU (2×LD50) of *H. parasuis* serotype 5 into the abdominal cavity 15 days after the second immunization. Within 5 h post-challenge, the mice exhibited lethargy and a significant reduction in activity. The PBS control group mice experienced rapid mortality within 2 days, with a mortality rate of 80%, and all succumbed within 3 days. The PLGA-OMP16 group exhibited a survival rate of 70%, while the OMP16 group showed a lower survival rate of 50%(Figure 5). The mice in the PLGA-OMP16 group displayed less severe clinical signs post-challenge and regained vitality after 2 days.

Upon analysis, the mice in the PBS control group that perished on the same day exhibited severe pulmonary blood stasis, partial spleen enlargement with blood stasis, cellulose exudate, and abdominal fluid. Furthermore, catarrhal and hemorrhagic lesions were evident in the small intestine. Conversely, the surviving mice examined after 7 days of challenge displayed no discernible organ lesions. The clinical symptoms observed in these resilient mice were mild and characterized only by a decrease in appetite and vitality.

### 3.6. Bacteriological Analysis

**Figure 6:**
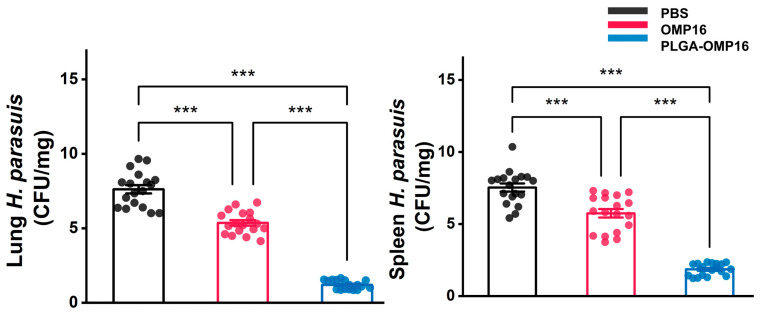
Plot of spleen and lung bacterial load. The bacterial load in the spleen and lung were evaluated by counting colonies of *H. parasuis* on TSA which was confirmed by a specific PCR method. The tissues were collected 24 h after the bacterial challenge. (*n* = 18 mice/group), ***, *p* < 0.001.

The bacterial loads in the spleen and lungs of the mice at 24 h post-infection were assessed to evaluate the clearance of *H. parasuis*. To confirm the presence of *Haemophilus parasuis*, three colonies from each plate were selected for PCR identification. The results demonstrated a significantly lower bacterial count in the PLGA-OMP16 group compared to the OMP16 group (*p* < 0.001) (Figure 6).

## 4. Discussion

In the past few decades, significant efforts have been dedicated to controlling *H. parasuis* infections with a particular focus on the development and utilization of vaccination, including subunit vaccines, which are considered to play an indispensable role [15,31]. In recent years, there has been a significant increase in research efforts directed towards developing subunit vaccines targeting *H. parasuis*. Given that many pathogens enter animals through mucosal surfaces, the development of mucosal vaccines is considered a promising strategy to combat *H. parasuis*. These vaccines have the potential to elicit strong immune responses locally and systemically at the site where the pathogen enters the body. However, it is important to note that to date no mucosal vaccine specifically targeting *H. parasuis* has been reported in the literature. This underscores the ongoing need for innovative approaches to vaccine development in order to effectively address the challenges posed by *H. parasuis* infections.

Prior research has shown that administering vaccines intranasally can effectively trigger the production of antibodies in various mucosal sites, including the upper and lower respiratory tract, salivary glands, and genital mucosa [32,33]. However, it is important to recognize that the nasal cavity is protected by a sophisticated mucosal barrier comprising cilia, mucous secretions, and tight junctions. This barrier plays a critical role in innate immunity and serves as a primary defense mechanism against invading pathogens [34]. This underscores the significance of understanding mucosal immunity and its implications for developing effective vaccination strategies against respiratory infections like those caused by *H. parasuis*. Therefore, it is essential for the delivery system of nasal vaccines to effectively address this obstacle. The PLGA particulate has emerged as one of the most widely utilized delivery systems capable of overcoming these barriers [35,36].

Based on our previous study, the OMP16 of *H. parasuis* has been demonstrated to be an effective antigen [25]. In this study, PLGA microspheres were utilized as carriers for the OMP16 antigens and were shown to effectively induce strong mucosal immunity. Particulate antigens have been found to be more efficient than soluble antigens in inducing systemic and mucosal immunity, possibly due to their enhanced endocytosis by mucosal-associated lymphoid tissue [37].

*H. parasuis*, being an extracellular pathogen, primarily relies on the humoral immune response, specifically antibodies, for protection against disease [38]. These antibodies play a crucial role in opsonizing H. parasuis bacteria and marking them for recognition and ingestion by alveolar macrophages, which subsequently leads to bacterial killing [8]. This process underscores the importance of understanding how antibodies contribute to immune defense against H. parasuis infections. In the present study, the PLGA-OMP16 microspheres induced a high level of IgG antibodies, which may aid in protecting animals against H. parasuis infections. PLGA nanoparticles are capable of continuously releasing entrapped antigens for extended periods in vitro. Using lower doses of these molecules would be advantageous, not only for minimizing the potential side effects often associated with adjuvant use, but also from an economic perspective [23].

There has been limited research on the roles of IgA antibodies and mucosal immune responses in the context of *Haemophilus parasuis* infections. The pathogenicity of H. parasuis is closely associated with its ability to evade the innate immune system by degrading IgA antibodies. Understanding these mechanisms is crucial, as they shed light on how H. parasuis establishes infection and evades host defenses, particularly within mucosal surfaces, where IgA plays a pivotal role in immune protection. Further investigation into these interactions is essential for developing strategies to enhance mucosal immunity and effectively combat H. parasuis infections [6]. In this study, the PLGA-Omp16 microspheres induced high levels of IgAs in the nasal, saliva, and BAL fluids. We hypothesize that this may contribute to bacterial clearance in the lungs and spleen. However, since the exact role of IgA was not specifically measured in this study, further research is needed to confirm this hypothesis.

Intranasal vaccination with OMP16 resulted in a robust local IgA antibody response in the nasal, salivary, and BAL secretions of the mice. This mucosal immunity effectively reduced the bacterial load in the spleen and lung following the challenge with *H. parasuis*. The administration of the PLGA-OMP16 microspheres via intranasal vaccination in mice effectively stimulated immune responses both locally and systemically. These findings indicate promising prospects for safeguarding against diseases caused by *H. parasuis* and mitigating its transmission and the emergence of variants. Additionally, intranasal vaccines have the potential to induce the formation of resident memory T and B cells, which further enhances the protection against local propagation and transmission of *H. parasuis*. This underscores the importance of exploring intranasal vaccination strategies to combat *H. parasuis* infections comprehensively.

The increase in IL-2 and IFN-γ concentrations suggests a possible activation of the type 1 helper T (Th1) cells by the PLGA-Omp16 microspheres. Moreover, the elevated IL-4 levels in the PLGA-Omp16 group indicate the activation of the type 2 helper T (Th2) cells. It is conceivable that both IFN-γ and IL-2 might stimulate the Th2 cells and promote the terminal differentiation of the IgA+ B cells into IgA-producing plasma cells. These findings suggest that the PLGA-Omp16 microspheres have the potential to modulate immune responses effectively. Further research is needed to explore their precise mechanisms and implications for mucosal immunity against *H. parasuis* infections [39]. The marked increase in IFN-γ and IL-2 production in mice treated with PLGA-Omp16 may contribute to the elevated levels of IgA. These findings indicate that PLGA-Omp16 has the potential to elicit a cell-mediated immune response. Taken together, these results further confirm the mixed Th1 and Th2 activation by PLGA-OMP16, which may provide comprehensive immune protections during *H. parasuis* invasions.

## 5. Conclusions

In conclusion, our findings illustrate that intranasal immunization using PLGA-OMP16 led to substantial IgG production and elicited a strong mucosal response. This vaccination approach demonstrates potential efficacy in offering sufficient protection against *H. parasuis* challenge, thereby presenting a promising avenue for advancing *H. parasuis* vaccine development.

## Data Availability

The raw data can be provided upon request. This study did not generate any code. All materials and reagents will be made available upon the execution of a material transfer agreement.
